# Tracing the Tiger: Population Genetics Provides Valuable Insights into the *Aedes* (*Stegomyia*) *albopictus* Invasion of the Australasian Region

**DOI:** 10.1371/journal.pntd.0002361

**Published:** 2013-08-08

**Authors:** Nigel W. Beebe, Luke Ambrose, Lydia A. Hill, Joseph B. Davis, George Hapgood, Robert D. Cooper, Richard C. Russell, Scott A. Ritchie, Lisa J. Reimer, Neil F. Lobo, Din Syafruddin, Andrew F. van den Hurk

**Affiliations:** 1 University of Queensland, St Lucia, Brisbane, Australia; 2 Commonwealth Scientific and Industrial Research Organisation (CSIRO) Ecosystem Sciences, Dutton Park, Brisbane, Australia; 3 Public Health Virology, Queensland Health Forensic and Scientific Services, Brisbane, Australia; 4 Queensland Tropical Population Health Network, Cairns, Australia; 5 Australian Army Malaria Institute, Brisbane, Australia; 6 The University of Sydney, Westmead Hospital, Sydney, Australia; 7 James Cook University, Cairns, Australia; 8 Papua New Guinea Institute of Medical Research, Madang, Papua New Guinea; 9 Eck Institute for Global Health, University of Notre Dame, Notre Dame, Indiana, United States of America; 10 Eijkman Institute for Molecular Biology, Jalan Diponegoro, Jakarta, Indonesia; The Pennsylvania State University, United States of America

## Abstract

**Background:**

The range of the Asian tiger mosquito *Aedes albopictus* is expanding globally, raising the threat of emerging and re-emerging arbovirus transmission risks including dengue and chikungunya. Its detection in Papua New Guinea's (PNG) southern Fly River coastal region in 1988 and 1992 placed it 150 km from mainland Australia. However, it was not until 12 years later that it appeared on the Torres Strait Islands. We hypothesized that the extant PNG population expanded into the Torres Straits as an indirect effect of drought-proofing the southern Fly River coastal villages in response to *El Nino*-driven climate variability in the region (via the rollout of rainwater tanks and water storage containers).

**Methodology/Principal Findings:**

Examination of the mosquito's mitochondrial DNA cytochrome oxidase I (COI) sequences and 13 novel nuclear microsatellites revealed evidence of substantial intermixing between PNG's southern Fly region and Torres Strait Island populations essentially compromising any island eradication attempts due to potential of reintroduction. However, two genetically distinct populations were identified in this region comprising the historically extant PNG populations and the exotic introduced population. Both COI sequence data and microsatellites showed the introduced population to have genetic affinities to populations from Timor Leste and Jakarta in the Indonesian region.

**Conclusions/Significance:**

The *Ae. albopictus* invasion into the Australian region was not a range expansion out of PNG as suspected, but founded by other, genetically distinct population(s), with strong genetic affinities to populations sampled from the Indonesian region. We now suspect that the introduction of *Ae. albopictus* into the Australian region was driven by widespread illegal fishing activity originating from the Indonesian region during this period. Human sea traffic is apparently shuttling this mosquito between islands in the Torres Strait and the southern PNG mainland and this extensive movement may well compromise *Ae. albopictus* eradication attempts in this region.

## Author Summary

The range of the Asian tiger mosquito *Aedes albopictus* is expanding globally, raising the threat of emerging and re-emerging arbovirus transmission risks, including chikungunya and dengue. Detection of *Ae. albopictus* in southern Papua New Guinea (PNG) in 1988 and 1992 placed it 150 km from mainland Australia. In 2004–05 it reached the islands in Australia's Torres Strait that separate the mainland from PNG. Suspecting a range expansion from PNG driven by human adaptation to climate variability, we employed population genetics methodologies to investigate possible origins of the introduced population, as well as population structure of previously existing populations from New Guinea. Mitochondrial cytochrome oxidase I sequences and 13 novel microsatellite markers revealed a clear genetic distinction between regional populations in PNG and the newly introduced population in the Torres Strait and Fly Region. The closest genetic relative to the introduced population was found in the Indonesian region to the west and it is now suspected that this species may have been brought into the Torres Strait by sea vessels involved in extensive illegal fishing activities during this period.

## Introduction

The Asian tiger mosquito *Aedes* (*Stegomyia*) *albopictus*, originally described by Skuse from Calcutta, India, in 1894, is considered native to the Southeast Asian region where the larvae are often found in forest tree holes – a characteristic that assists its current global expansion via rapid adaptation to human-made container habitats [1], [2], [3]. This global expansion is also driven by human behavior, often facilitated by the transport of used tyres that contain desiccation-resistant eggs or, in some cases, by the movement of the containers themselves [4], [5]. Prior to the 1980s, *Ae. albopictus* had spread to several islands in the Indian Ocean, as well as to the Hawaiian Islands in the Pacific [6]. It was discovered in Albania in Europe in 1979 [7], and has also established in both North [8] and South America [9], in Africa in 1992 [10], and in southern Europe [11]. It is currently expanding into over 20 European countries [2].

Alongside this species' global expansion, its status as a vector of human pathogens is also of increasing concern. As a laboratory vector of over 25 arboviruses, its role in arbovirus transmission cycles has mostly been secondary to other incriminated vectors [12], [13], [14]. In the absence of the primary dengue vector, *Aedes* (*Stegomyia*) *aegypti*, *Ae. albopictus* has been the epidemic vector of dengue viruses in Hawaii, Macao and China [14], [15], [16], [17]. In 2005, it was implicated as the epidemic vector during a resurgence of chikungunya (CHIKV), an alpha virus clinically similar to dengue, in the Indian Ocean and in Italy [17], [18], [19]. Subsequent studies revealed that *Ae. albopictus* is highly susceptible to the CHIKV, with the species not only responsible for these outbreaks but also able to transmit the virus after only two days [17], [20], [21].

**Figure 1:**
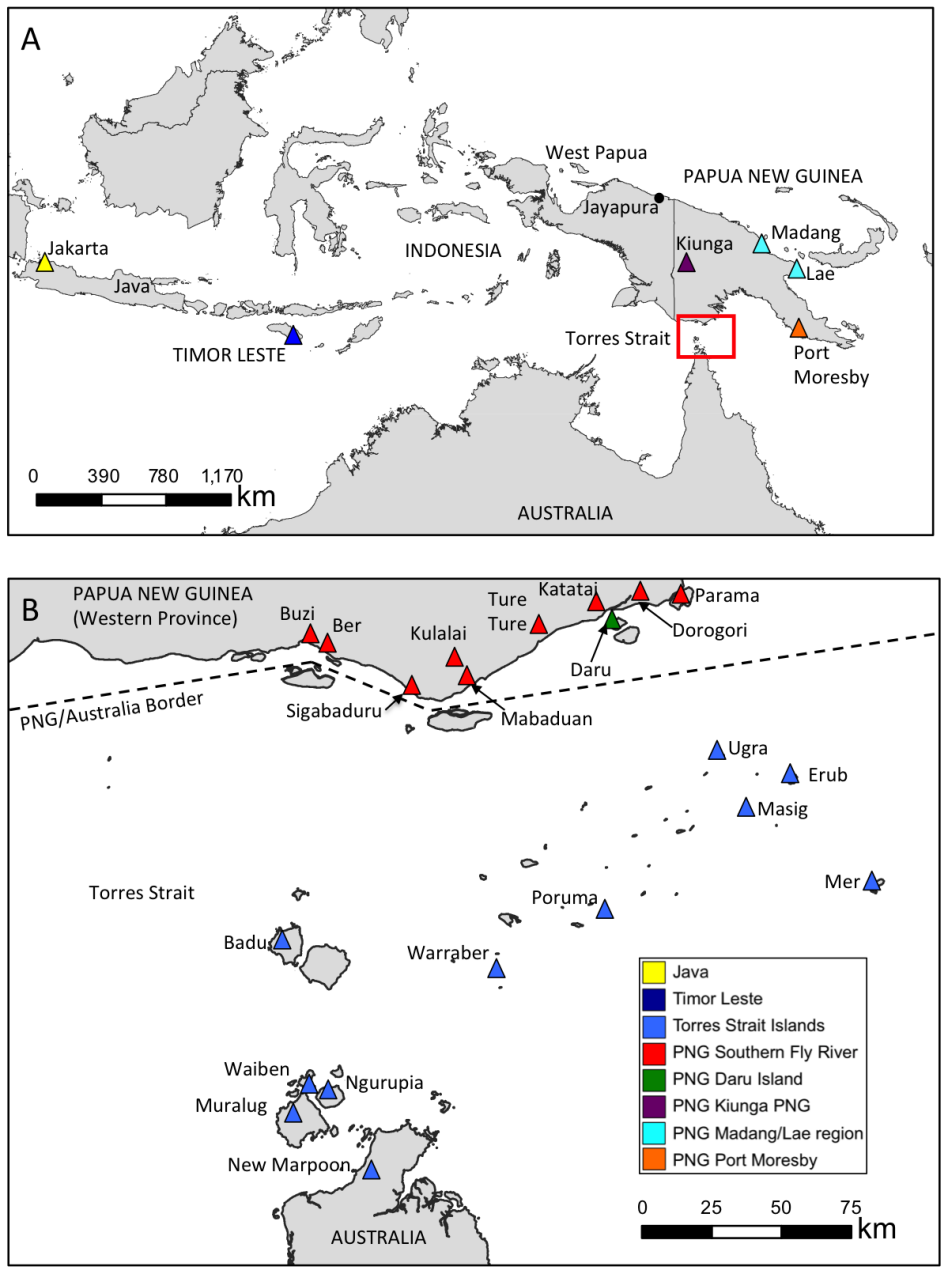
Map of the region under study and mosquito collection sites (Panel A). An expanded view of the region inhabited by the recently introduced *Ae. albopictus* population (red box) with collection sites indicated in Panel B. Colored triangles correspond to sample locations.

In the Australasian region, *Ae. albopictus* was first detected in 1963 in Jayapura on the West Papua Province of Indonesia (see Fig. 1A). Subsequent surveys during the early 1970s confirmed its presence in northern Papua New Guinea (PNG) near Madang [22], [23]. By 1980 it had arrived in southern PNG's Port Moresby (PNG's capital), and moved eastwards into Bougainville Province and the Solomon Islands [24], [25]. Its detection in southern PNG's Western Province southern Fly River coastal fringe in 1988 (see Fig. 1B), combined with surveys in 1992 revealing it on Daru Island in the northern Torres Strait region and in Kiunga Port over 700 km up the Fly River, established beyond doubt that the species was extant just 150 km from mainland Australia's Cape York [24], [26]. Despite there being at least 28 collections of *Ae. albopictus* at six Australian seaports, this species has not yet established on Australia's mainland [27].

In 2005, *Ae. albopictus* was detected on Masig Island in the central Torres Strait Islands and molecular identification of previously collected *Ae. albopictus* larvae [28] (which discriminated it from local *Ae. scutellaris* species), dated its arrival to 2004. Subsequent surveys in the Torres Strait revealed its presence on 10 of the 17 inhabited islands [29]. Considering the potential of both the human health and societal (nuisance) impacts of *Ae. albopictus* establishing on mainland Australia, the obvious question was why *Ae. albopictus* had only expanded into the Torres Strait Islands in 2004–05 when it was known to have been extant on Daru Island (northern Torres Strait) and in Kiunga in 1992 – 12 years earlier? A number of potential sociological and ecological factors may have contributed to the mosquitoes' proliferation and led to its dramatic expansion into the Torres Strait islands in the mid-2000s. For example, the increase in human-made water storage containers and sundry smaller discarded disposable containers may have served as potential larval habitats, leading to a population expansion.

The discovery of *Ae. albopictus* in the Torres Strait in the mid 2000s led to the question of whether recent adaptation to climatic variability had played a role in its expansion – as we had suggested in earlier work on other container inhabiting *Aedes* species in this region [30]. The 1997–98 *El Nino* conditions contributed to the worst drought in PNG for 100 years: traditional groundwater supplies were greatly affected, either drying up or becoming contaminated [31]. As local springs and streams dried up, it became necessary for villages to store water in large containers including 220 L (44 gal) drums and rainwater tanks – both of which provide highly productive larval sites for container-inhabiting mosquito species [32]. As part of an international aid response, AusAID funded and transported 9,000 L polypropylene rainwater tanks and 200 L water containers to the southern Fly River region villages immediately adjacent to the Torres Strait in a project completed in 2002 [33], [34]. This human adaptation to climate variability may have provided abundant productive larval sources for the population of *Ae. albopictus* already present in PNG, leading to its rapid population expansion and a subsequent spillover into the Torres Strait Islands. Once *Ae. albopictus* was established on the islands, the continuous human ocean traffic would have rapidly shuttled mosquitoes through the region. Thus our working hypothesis was that climate variability driven by the 1997–98 *El Nino* resulted in water storage management changes in PNG's southern Fly River coastal villages and was indirectly responsible for the invasion of a local *Ae. albopictus* population through the Torres Strait Islands.

In this study, we use extensive regional mosquito collections and population genetics methodologies to investigate the origins and dynamics of the introduction of *Ae. albopictus* into and through the Torres Strait islands, as well as the population structure of this species throughout PNG. The maternally inherited mitochondrial DNA (mtDNA) cytochrome oxidase I (COI) is used as both a population genetics marker and a proxy for female movement between islands and southern PNG villages. The rationale here is that the DNA sequence of each female is (barring mutation) identical to that of her offspring, providing insights into the dynamics and diversity of the females' contribution to each population. This proxy would ultimately be an underestimate of movement as different females of the same sequence cannot be distinguished. Additionally, we developed and ran 13 microsatellites markers that permitted the evaluation of the nuclear background of these mosquitoes.

## Materials and Methods

### Mosquito samples

Container-inhabiting mosquitoes were collected from throughout the Torres Strait and PNG's southern Fly River region villages by Queensland Health between 2004 and 2010 (Tables 1 and 2, and Figure 1). Populations of *Ae. albopictus* collected in 1992 from Daru Island (northeastern Torres Straits) and the Kiunga Port area in Western Province were provided by the Australian Defence Force. When samples were collected from private residences, permission was granted prior to entry. In most cases larvae were sampled from different containers at each location and preserved in 70% ethanol, and in some cases adults were collected. In many cases only a few individuals were collected at each location in order to reduce the chance of sampling siblings (as larvae in the same container). Larvae were initially identified as *Ae. albopictus* using the morphological keys of [35] and then by either real-time PCR assays [36] or by a PCR-restriction digest procedure [28] to distinguish them from endemic members of the *Aedes* (*Stegomyia*) *scutellaris* taxonomic group.

**Table 1:** Summary of mtDNA COI study collection sites and haplotype distribution.

Region	Site	Year	Haplotypes																
			1	2	3	4	5	6	7	8	9	10	11	12	13	14	15	16	Totals
**PNG Southern Fly**	Buzi	2007						1					1						2
	Ber	2007						3					4						7
	Sigabaduru	2007						7					1						8
	Mabaduan	2007						7					6	7			2		22
	Ture Ture	2007						5					1						6
	Katatai	2007						3					2			1			6
	Dorogori	2007						1				2		1					4
	Parama	2007						5				3		4					12
**Torres Strait Nth**	Eurb	2006						3				2	2	1		4			12
	Ugar	2006						8				1					1		10
	Masig	2006, 2007						4					4	2			4		14
	Mer	2007						2											2
**Torres Strait Central**	Poruma	2007										8		1					9
	Mabuiag	2007											1				2		3
	Badu	2007						2			1	4	1						8
	Moa	2007						1					7						8
	Warraber	2007						2						2			6		10
**Torres Strait South**	Waiben	2010											4				3		7
	Ngarupai	2010	1					16	1				6		1		2		27
	Muralag	2010						2						1					3
**Cape York**	New Marpoon	2010															2		2
**PNG**	Daru	1992, 2008	29	1		1	2						1	1					35
**PNG**	Kiunga	1992	34	1									3						38
**PNG**	Port Moresby	1997, 1998	41		1			12		1			3						58
**PNG**	Madang/Lae	2011/1996	29					10											39
**Indonesia**	Timor Leste	2001											23	1					17
**Indonesia**	Jakarta	2011											7					1	8
**Totals**			134	2	1	1	2	94	1	1	1	20	70	21	1	5	22	1	377

**Table 2:** Mosquito sampling summary for microsatellite study.

Region	Site	Year	n
**PNG Southern Fly**	Kulalai	2007	2
	Sigabaduru	2007	1
	Katatai	2007	2
	Mabudauan	2007	7
**Torres Strait North**	Masig	2006/2007	21
	Mer	2007	6
**Torres Strait Central**	Mabuiag	2007	10
	Warraber	2007	8
**Torres Strait South**	Waiben	2010	3
	Ngarupai	2010	7
	Muralag	2010	5
**PNG**	Daru	1992, 2008	26
	Kiunga	1992	20
	Port Moresby	1997, 1998	29
	Madang/Lae	2011, 1996	33
**Indonesian region**	Timor Leste	2001	10
	Jakarta	2011	9

### mtDNA sequencing and analyses

Specimens identified as *Ae. albopictus* had genomic DNA extracted using a salt extraction method [37]. For PCR amplification of a 445 bp (final edited product size) region of the mtDNA cytochrome oxidase 1 (COI), the forward primer 5′ CAY CCT GGT ATA TTT ATT GG ′3 and reverse primer 5′AAT TAA AAT ATA AAC TTC TGG were modified from [38]. The reaction was carried out in 0.2 ml well PCR plates (Astral Scientific) using 25 µl final volume and oil overlay (single drop). Final PCR mixture contained 16.6 mM [NH4]_2_SO4, 67 mM Tris-HCl pH 8.8 (at 25°C), 0.45% Triton X-100, 0.2 mg/ml gelatin, 1.5 mM MgCl, 0.2 mM of each dNTP, 0.4 µM of each primer. One unit of *Taq* polymerase (Bioline) and 2–10 ng of purified genomic DNA (1 µl of gDNA) were used per reaction. Cycling (MJ research PTC200 or a BioRad C-1000 thermal cycler) was 94°C for 3 min followed by 30 cycles of 94°C for 1 min, 40°C for 1 min, and 72°C for 1 min using minimum transition times between steps. The PCR products were visualized on a 1% agarose gel containing 0.5 µg/ml ethidium bromide and visualized at 312 nm. PCR product purification was via QIAGEN (QIAquick) PCR purification columns using manufactures recommendations.

All sequences were edited and aligned using the Geneious software [39]. To examine phylogeographic relationships, we constructed maximum parsimony haplotype networks in TCS 1.21 [40] under a 95% connection limit. Pair-wise *F*_ST_ values were estimated in Arlequin version 3.5 (distance method) [41] to assess levels of differentiation between the regions for the COI locus: regions were designated Torres Strait Islands (excluding Daru Island), southern Fly region, Daru Island, Kiunga, Port Moresby, Madang/Lae Region, Timor Leste and Jakarta. The significance levels of *F*_ST_ comparisons were assessed using permutation tests (1,023 permutations per comparison), also implemented in Arlequin. DnaSP 5 [42] was used to estimate haplotype diversity and nucleotide diversity within regions. We performed Tajima's D and Fu's Fs tests of neutrality for the COI data per population in the program Arlequin.

### Microsatellite development and population genetics analyses

Candidate microsatellite markers were isolated from Roche GS FLX 454 sequencing data (1/16 plate - 25,000 reads at ∼400 bp length) generated from genomic DNA of *Ae. albopictus* and performed by Macrogen (Korea). To design primers for microsatellite loci, we ran the resultant data through the program msatcommander [43]. We used this program to find primers for dinucleotide, trinucleotide and tetranucleotide repeats, and allowed the program to design primers with a melting temperature in the range of 50–62°C with a GC content between 30 and 70 percent. Long polynucleotide repeats (>5 bp) within sequences to be amplified were avoided and duplicate markers (i.e. primers designed for sequence analogues) were excluded. Screening of candidate markers involved PCR amplification of a subset of samples using standard primers and visualization of products on 1% ethidium bromide stained agarose gels. Positive product primer sets were re-amplified with M13 labeled forward primers and dyes (VIC, FAM, PET and NED) and standard reverse primers. The final PCR mixture contained 1× My*taq* buffer (Bioline)(containing pre-optimized concentrations of MgCl and dNTPs), 0.4 µM of each primer, 0.5–1.0 unit of My*Taq* polymerase (Bioline) and 5.0–10.0 ng of extracted genomic DNA (1 µl of extraction). The cycling involved an initial denaturation of 95°C for 3 min, then 13 cycles of 95°C for 30 s, 56°C for 40 s with a gradient decrease of 0.5°C/cycle, and 72°C for 30 s, followed by 25 cycles of 95°C for 30 s, 50°C for 40 s and 72°C for 30 s, and a final 72°C for 5 min using minimum transition times. M13 labeled products for 13 microsatellite markers (see Table 3 for details) that generated clean peaks and that amplified consistently were purified using ExoSap (Antarctic phosphatase and Exonuclease I-New England Biolab) and were sent to Macrogen (Macrogen, Geumchun-gu, Seoul, Korea) for genotyping. We attempted to genotype 199 individuals sampled from the Torres Strait Islands, New Guinea, Timor Leste and Jakarta (see Table 2 for sampling information).

**Table 3:** Microsatellite primer and allelic information.

	#Alleles	Size Range (bp)	Forward	Reverse	Genbank
Alb-di-4	14	166–200	TGGCGACCTATTATACCCGC	CAACTCGTTCCTTGACCGTG	KF146971
Alb-di-6	11	268–290	TCTTCATCTACGCTGTGCTC	GACGCCAATCCGACAAAGTC	KF146972
Alb-tri-3	9	123–153	AGATGTGTCGCAATGCTTCC	GATTCGGTGATGTTGAGGCC	KF146973
Alb-tri-6	17	164–219	AGCACGAGTACAGAATGTGC	TGGCCTCCTACCGTTTATCTG	KF146974
Alb-tri-18	10	250–280	ACACAATTGCCGTTCAGCTC	CGTCTAATAGCTCCGGTCCC	KF146975
Alb-tri-20	15	165–201	GTGCCGTTGATCATCCTGTC	TCCAGCACCGTGAGTAATCC	KF146976
Alb-tri-21	17	137–206	AGGGCTTCAATGGGTCTCTC	TGGTTATTAATACGGCGAGGC	KF146977
Alb-tri-25	8	257–278	CCAACCAACAACCCAGGAAC	TACGATGCGCAACCATCATC	KF146978
Alb-tri-33	11	137–182	GGCTGCTGTTGTTGGTACG	CACGTTCAATCACCGGTTCC	KF146979
Alb-tri-41	8	134–155	GATCGATTTGGGAGCTTCTG	GAACCTCTTCTCGCTTGGCT	KF146980
Alb-tri-44	10	173–212	CACTCGCGCGTGTTCTTC	GACGCACCATCAGCATCATC	KF146981
Alb-tri-45	9	120–150	TTTCAGCTCGGTGTTATGGC	TGATGTTGATGATGATGACTACGA	KF146982
Alb-tri-46	10	158–192	TTCACAACATACGGAATCGC	GGTCCGGTGTAATAGCCTCC	KF146983

Alleles for each marker were scored manually in the program GeneMarker [44]. We checked for the possible presence of null alleles for each marker at a population level (based on the regions: Torres Strait, Fly Region, Daru, Kiunga, Madang, Port Moresby, Timor Leste and Jakarta) using the program MICRO-CHECKER [45]. Using these same population definitions, we checked for HWE, as well as calculating observed (Ho) and expected (He) heterozygosity in the program GenAlEx, v6 [46] and the program GenoDive [47] was used to calculated Fis for each population (Table 4 contains details of Null Alleles, HWE, etc).

**Table 4:** Characteristics of microsatellite markers per population.

	Torres Strait (n = 60)		Fly Region PNG (n = 12)		Daru (n = 26)		Kiunga (n = 20)	
Locus	*HWE, Null, % missing*	*[Na, Fis] (Ho, He)*	*HWE, Null, % missing*	*[Na, Fis] (Ho, He)*	*HWE, Null, % missing*	*[Na, Fis] (Ho, He)*	*HWE, Null, % missing*	*[Na, Fis] (Ho, He)*
**DI-4**	n, y, 0	[5, 0.505] (0.233, 0.465)	n, y, 8	[5, 0.623] (0.182, 0.446)	y, n, 4	[5, 0.110] (0.520, 0.571)	n, y, 10	[3, 0.585] (0.222, 0.512)
**DI-6**	n, y, 5	[8, 0.491] (0.368, 0.700)	y, y, 0	[6, 0.675] (0.250, 0.715)	n, y, 0	[7, 0.355] (0.385, 0.581)	n, n, 5	[4, 0.324] (0.368, 0.526)
**TRI-3**	n, y, 0	[7, 0.522] (0.300, 0.661)	y, n, 0	[3, 0.267] (0.417, 0.538)	y, n, 0	[5, 0.166] (0.538, 0.631)	y, y, 0	[4, 0.575] (0.300, 0.679)
**TRI-6**	n, n, 10	[12, 0.083] (0.630, 0.680)	y, n, 8	[5, 0.200] (0.636, 0.752)	n, y, 16	[6, 0.512] (0.364, 0.720)	n, y, 30	[6, 0.644] (0.286, 0.755)
**TRI-18**	n, y, 22	[6, 0.716] (0.213, 0.737)	y, y, 50	[4, 0.792] (0.167, 0.681)	y, n, 4	[6, 0.248] (0.520, 0.674)	y, y, 35	[7, 0.544] (0.385, 0.793)
**TRI-20**	n, y, 0	[13, 0.421] (0.483, 0.825)	y, y, 8	[7, 0.459] (0.455, 0.785)	y, n, 0	[8, 0.012] (0.769, 0.763)	n, y, 10	[4, 0.812] (0.111, 0.562)
**TRI-21**	n, y, 22	[9, 0.650] (0.277, 0.776)	n, y, 17	[7, 0.744] (0.200, 0.805)	n, y, 8	[5, 0.939] (0.042, 0.652)	n, n, 30	[3, 0.235] (0.286, 0.357)
**TRI-25**	y, n, 0	[5, 0.166] (0.517, 0.614)	y, n, 8	[7, 0.221] (0.545, 0.661)	y, y, 0	[5, 0.361] (0.500, 0.763)	y, n, 75	[3, 0] (0.600, 0.540)
**TRI-33**	y, n, 0	[7, 0.093] (0.683, 0.747)	y, n, 8	[4, 0.277] (0.545, 0.711)	n, n, 4	[7, 0.199] (0.560, 0.682)	y, n, 5	[6, 0.238] (0.579, 0.735)
**TRI-41**	n, y, 3	[6, 0.425] (0.345, 0.593)	y, n, 8	[4, 0.469] (0.273, 0.479)	y, n, 4	[2, 0.134] (0.440, 0.497)	y, n, 5	[2, 0.345] (0.316, 0.465)
**TRI-44**	n, y, 8	[7, 0.433] (0.436, 0.760)	y, n, 17	[5, −0.091] (0.800, 0.700)	y, n, 0	[5, −0.004] (0.654, 0.639)	y, y, 5	[5, 0.396] (0.368, 0.587)
**TRI-45**	y, n, 2	[6, 0.013] (0.542, 0.545)	y, y, 8	[7, 0.394] (0.455, 0.702)	y, n, 0	[6, −0.122] (0.846, 0.741)	y, n, 0	[3, 0.150] (0.550, 0.629)
**TRI-46**	y, n, 3	[6, 0.002] (0.621, 0.617)	y, n, 8	[4, 0.161] (0.545, 0.616)	n, y, 12	[3, 0.588] (0.261, 0.612)	y, n, 30	[3, 0.444] (0.286, 0.487)

	Madang (n = 33)		Port Moresby (n = 29)		Timor Leste (n = 10)		Jakarta (n = 9)	
Locus	*HWE, Null, % missing*	*[Na, Fis] (Ho, He)*	*HWE, Null, % missing*	*[Na, Fis] (Ho, He)*	*HWE, Null, % missing*	*[Na, Fis] (Ho, He)*	*HWE, Null, % missing*	*[Na, Fis] (Ho, He)*
**DI-4**	n, y, 0	[7, 0.543] (0.273, 0.583)	y, y, 4	[4, 0.475] (0.214, 0.397)	y, n, 0	[4, 0.463] (0.200, 0.345)	y, n, 0	[4, −0.247] (0.667, 0.512)
**DI-6**	n, y, 0	[7, 0.291] (0.515, 0.712)	y, y, 0	[4, 0.496] (0.345, 0.666)	n, y, 0	[6, 0.755] (0.200, 0.745)	y, n, 0	[3, −0.032] (0.222, 0.204)
**TRI-3**	n, y, 0	[7, 0.277] (0.485, 0.657)	y, y, 0	[5, 0.284] (0.552, 0.753)	n, n, 0	[4, 0.550] (0.300, 0.615)	y, y, 11	[4, 0.844] (0.125, 0.711)
**TRI-6**	n, y, 0	[9, 0.469] (0.424, 0.781)	y, y, 0	[9, 0.458] (0.448, 0.806)	y, y, 10	[6, 0.484] (0.444, 0.790)	y, y, 0	[7, 0.508] (0.444, 0.827)
**TRI-18**	y, n, 0	[7, 0.144] (0.667, 0.765)	y, n, 4	[8, 0.212] (0.643, 0.798)	y, y, 0	[5, 0.628] (0.300, 0.740)	y, y, 22	[4, 0.829] (0.143, 0.724)
**TRI-20**	n, n, 0	[10, 0.171] (0.667, 0.790)	n, n, 0	[8, 0.076] (0.655, 0.696)	y, n, 0	[7, 0.027] (0.800, 0.780)	y, n, 0	[5, −0.028] (0.778, 0.716)
**TRI-21**	n, y, 3	[9, 0.581] (0.313, 0.728)	n, y, 0	[6, 0.647] (0.276, 0.759)	y, n, 40	[4, 0.592] (0.333, 0.708)	y, n, 0	[3, 0.273] (0.333, 0.426)
**TRI-25**	y, n, 0	[5, 0.153] (0.606, 0.703)	n, n, 4	[6, 0.090] (0.679, 0.732)	y, n, 0	[6, 0.176] (0.600, 0.685)	y, n, 11	[5, 0.103] (0.625, 0.648)
**TRI-33**	y, n, 0	[7, 0.070] (0.636, 0.673)	y, n, 0	[5, 0.190] (0.552, 0.667)	y, n, 0	[4, 0.069] (0.600, 0.610)	y, n, 0	[5, −0.013] (0.556, 0.519)
**TRI-41**	n, y, 0	[4, 0.501] (0.273, 0.534)	n, y, 4	[6, 0.265] (0.500, 0.665)	y, n, 10	[4, 0.489] (0.333, 0.599)	y, n, 0	[5, 0.461] (0.333, 0.568)
**TRI-44**	n, n, 0	[5, 0.098] (0.485, 0.528)	n, n, 0	[6, 0.157] (0.448, 0.521)	y, n, 0	[5, −0.008] (0.700, 0.660)	y, n, 11	[3, −0.105] (0.375, 0.320)
**TRI-45**	y, n, 0	[5, −0.120] (0.758, 0.668)	y, n, 0	[5, 0.073] (0.724, 0.766)	y, n, 0	[5, −0.220] (0.800, 0.630)	y, n, 0	[3, −0.212] (0.556, 0.438)
**TRI-46**	n, y, 0	[7, 0.445] (0.333, 0.587)	n, y, 10	[5, 0.632] (0.269, 0.709)	y, n, 0	[5, 0.085] (0.600, 0.620)	y, n, 0	[4, 0] (0.778, 0.735)

The Bayesian program STRUCTURE, v. 2.3.2 [48], [49], was used to infer the most likely number of genetically distinct groups (*K*) in the region sampled, based on the microsatellite data. STRUCTURE was run using the admixture model, and using sampling locations as priors. Including information on sampling locations in STRUCTURE analyses has been shown to be useful for detecting subtle genetic structure, without detecting structure that is not present [48]. Due to the potential presence of null alleles at a number of markers in some populations, we used a dominant marker model in STRUCTURE (as recommended in the user manual). STRUCTURE was run for five iterations of *K* = 2 to *K* = 8, for a total of 1 million generations per iteration with a burn-in of 200 000 iterations. STRUCTUREHARVESTER, a program that implements the Evanno et al. Delta K method [50], [51], was then used to infer the most likely value of *K* and CLUMPP v. 1.1.2 [52] was used to average the results of the replicates for *K* = 5 (the most likely value based on the Delta K method). We used the Greedy algorithm in CLUMPP with 1000 repeats. The output from CLUMPP was run through DISTRUCT [53], which provides more flexibility in generating figures than STRUCTURE. Additionally, the program GENETIX v. 4.05 [54] was used to perform a Factorial Correspondence Analysis (FCA). FSTAT v2.9.3 [55] was used to test for linkage disequilibrium between loci, and finally Arlequin v.3.5 [41] was used to estimate pair-wise *F*_ST_ values between the eight populations defined above.

## Results

### Mitochondrial DNA

A total of 16 mtDNA COI haplotypes were identified from 377 individuals (haplotype diversity Hd = 0.769) collected throughout the southern Fly River villages, the Torres Strait islands, north and south PNG, Timor Leste and Jakarta – 10 of these haplotypes were present in the Torres Strait Islands and southern Fly River region (see Table 1 and Fig. 1 for details on collections and mtDNA haplotype occurrence). All DNA sequences are available through GenBank (KC572145 - KC572496, KF042861-KF042885) and all tests of neutrality (Tajima's D and Fu's Fs) were non-significant. Haplotype diversity varied for each region with the Torres Strait Islands (122 individuals, 10 haplotypes, Hd = 0.801) and southern Fly River region (60 individuals, 6 haplotypes, Hd = 0.649) having substantially higher haplotype diversities than the PNG populations from Daru Island (35 individuals, 6 haplotypes, Hd = 316), Kiunga (38 individuals, 3 haplotypes, Hd = 0.198), Port Moresby (58 individuals, 5 haplotypes, Hd = 0.462) and Madang/Lae Region (39 individuals, 2 haplotypes, Hd = 0.391) as well as those from Timor Leste (17 individuals, 2 haplotypes, Hd = 0.118) and Jakarta (8 individuals, 2 haplotypes, Hd = 0.250).

**Figure 2:**
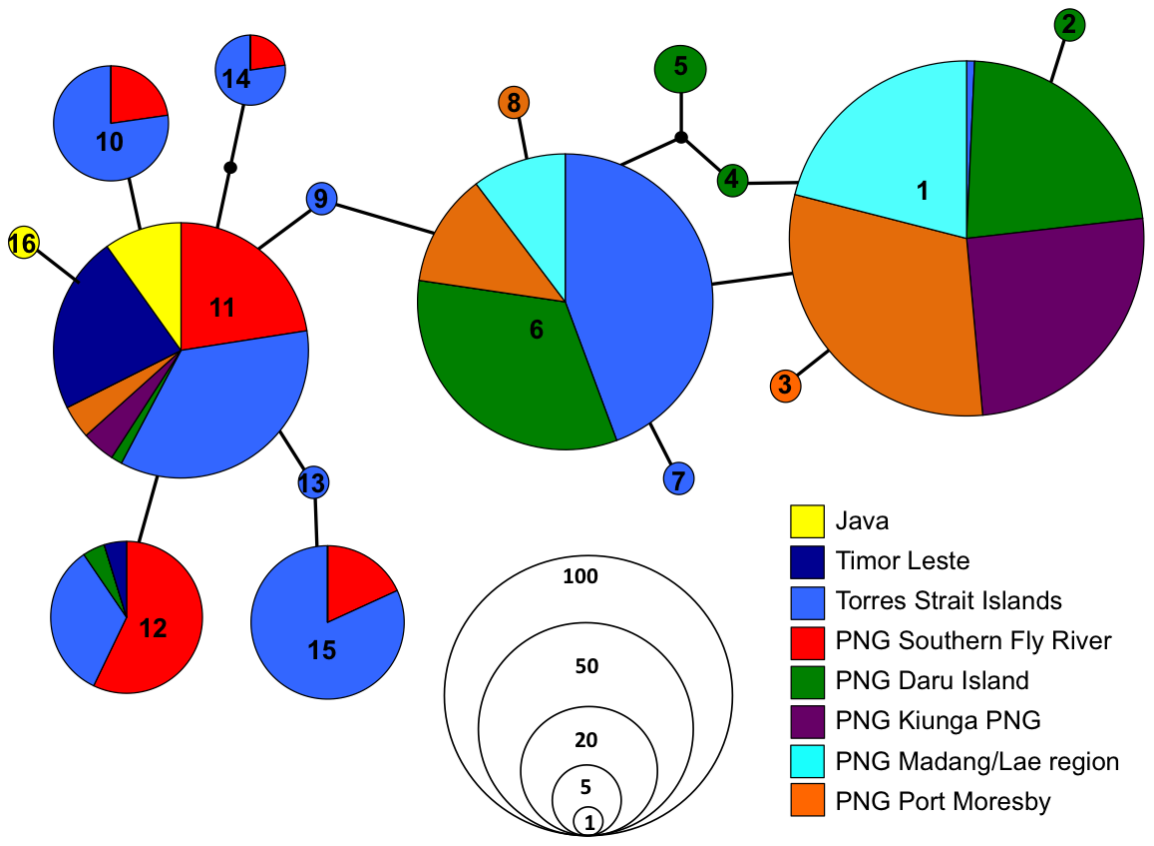
Mitochondrial COI haplotype network showing the 16 haplotypes identified throughout PNG and the Australian region. Haplotypes are colored by region and the size of their circle is proportional to number of individuals showing that haplotype sequence with each connection a single mutational step.

The COI haplotype network (Figure 2) suggests that there is some mitochondrial genetic structure between regions, with one of the most common haplotypes (H1) sampled found predominantly in the populations extant in PNG (the Madang/Lae region, Port Moresby, Kiunga and Daru Island – with Daru collections from both 1988 and 2008), and only being sampled once (in one individual) in the Torres Strait Islands. The other common PNG haplotype (H6) was sampled at relatively high frequency in the Torres Strait. Two other haplotypes (H11 and H12) that were sampled relatively commonly in both the Torres Strait and in the Fly Region of PNG were also sampled in Daru. Haplotype 11 was also the most predominant haplotype sampled in both of the Indonesian populations (Jakarta and Timor Leste), with Timor Leste also sharing H12 and Jakarta possessing one other private haplotype (H16). This suggests that there is a close affinity between Indonesian populations and those found in the Fly Region of PNG as well as those in the Torres Strait. Six private haplotypes were sampled in the Fly Region/Torres Strait (H7, H9, H10, H13, H14, H15), three of which were only found singly in Torres Strait (H7, H9, H13) and there were 5 private haplotypes sampled in PNG; H2, H4 and H5 found only in Daru; with H3 and H8 found only in Port Moresby.

Mitochondrial COI pair-wise *F*_ST_ relationships and significance comparisons supported the presence of structure between populations (see Table 5). Again the PNG Southern Fly Region and Torres Strait populations appeared highly distinct from the PNG populations with high and significant *F*_ST_ values between the populations (roughly between 0.4 to 0.5). The *F*_ST_ value between Torres Strait and Fly Region populations is significant but small (0.043), and most comparisons between PNG populations are non-significant (all *F*_ST_<0.1). Indonesian populations are not significantly differentiated from each other but have significant *F*_ST_ values in all other comparisons, with the Torres Strait populations being the most closely related to them, followed by the Fly Region and then by PNG populations.

**Table 5:** Pairwise COI *F*_ST_ values (bold = non-significant).

	Torres Strait	Fly Region	Daru	Kiunga	Port Moresby	Madang	Timor Leste
Fly Region	0.04294						
Daru	0.46207	0.46889					
Kiunga	0.47707	0.50317	**−0.00658**				
Port Moresby	0.44540	0.43966	**0.02975**	0.04464			
Madang	0.46563	0.47669	**0.03726**	0.06915	**−0.01029**		
Timor Leste	0.12338	0.30382	0.81124	0.86803	0.80658	0.89127	
Jakarta	0.09859	0.26626	0.77880	0.84574	0.78493	0.87307	**0.02812**

### Microsatellites

A total of 199 individuals were genotyped for the 13 microsatellites (see Table 2 for mosquito sampling). Putative null alleles were found at some loci in some populations and tests for Hardy Weinberg equilibrium revealed that some loci did not meet the expectations of this model (see Table 4 for detailed information), but no evidence of linkage disequilibrium between loci was found. The overall number of alleles per locus ranged from 8 to 17 (Table 3). The inbreeding coefficients (F_IS_) of the majority of loci were positive, and observed heterozygosity was less than expected heterozygosity in most cases, indicating an excess of homozygote genotypes at most loci (see Table 4). Although *F*_ST_ values are generally smaller for the microsatellite data than for the mitochondrial data (Tables 5 & 6), all microsatellite based pair-wise *F*_ST_ values between populations were significant, with the exception of the Torres Strait – Fly Region comparison (*F*_ST_ = 0.00421, Table 6). Low *F*_ST_ values were found between PNG populations, as well as between Torres Strait/Fly Region populations and those from Indonesia, providing further evidence of close affinities between these populations.

**Table 6:** Pairwise *F*_ST_ msats: 13 loci (bold = non-significant).

	Torres Strait	Fly Region	Daru	Kiunga	Madang	Port Moresby	Timor Leste
Fly Region	**0.00421**						
Daru	0.15061	0.13217					
Kiunga	0.14283	0.16632	0.07760				
Madang	0.15134	0.13189	0.03805	0.07871			
Port Moresby	0.12791	0.11830	0.09477	0.05612	0.08168		
Timor Leste	0.10243	0.07446	0.15200	0.11821	0.15745	0.08435	
Jakarta	0.13908	0.12848	0.25316	0.26416	0.24757	0.20855	0.11396

**Figure 3:**
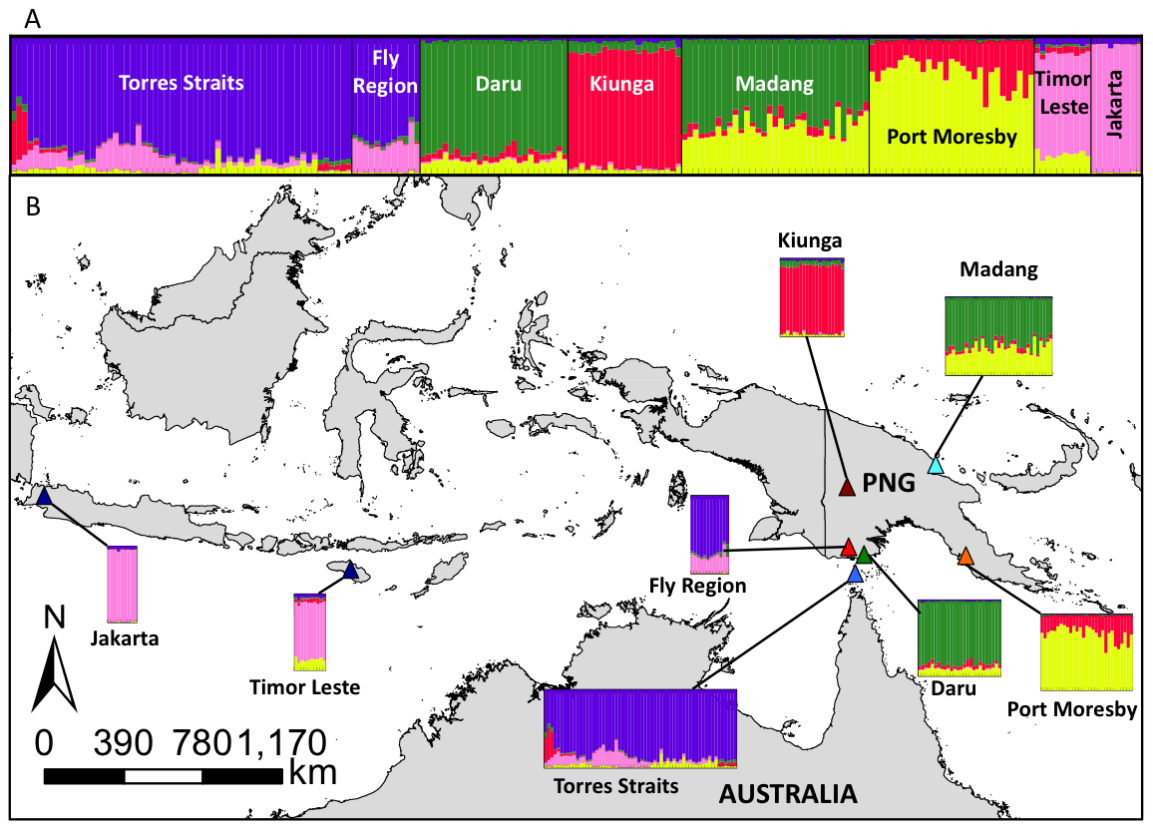
Panel A: Microsatellite Bayesian Structure Plot for *Ae. albopictus*. Thirteen microsatellite markers were run on 199 *Ae. albopictus* individuals assuming five populations (*K* = 5). Each bar represents an individual with the color of the bar the probability of the individual belonging to a genetic population or cluster. **Panel B:** The structure plot results (*K* = 5) integrated into a map of the region with the locations of each population shown.

The most likely number of genetic clusters (*K*) inferred by STRUCTURE HARVESTER was *K* = 5 [50], [51]. The bar plot generated in STRUCTURE (Fig. 3) suggests five populations (Figure 3) with three historically extant populations within PNG that may have experienced various levels of admixture, and one distinct population encompassing the Torres Strait Islands and the southern Fly River region (purple). An additional population was found in Indonesia (pink), and bar-plots suggest some similarity of these populations to some individuals in the Torres Strait and Fly Region. The populations from Daru Island (collected in 1992 and 2008), which sits geographically adjacent to the southern Fly River coastal region, are clearly differentiated from the introduced populations, with all individuals from these regions being assigned with high probability to a single population (green). Samples from Kiunga are assigned with high probability to a distinct cluster (red) to which individuals from Port Moresby are also partially assigned, although these (Port Moresby) individuals are also assigned to another cluster (yellow) with higher probability. Individuals from Madang in northern PNG are assigned with highest probability to the green cluster (mostly found in Daru) and with a lower probability to the yellow cluster (mostly found in Port Moresby).

**Figure 4:**
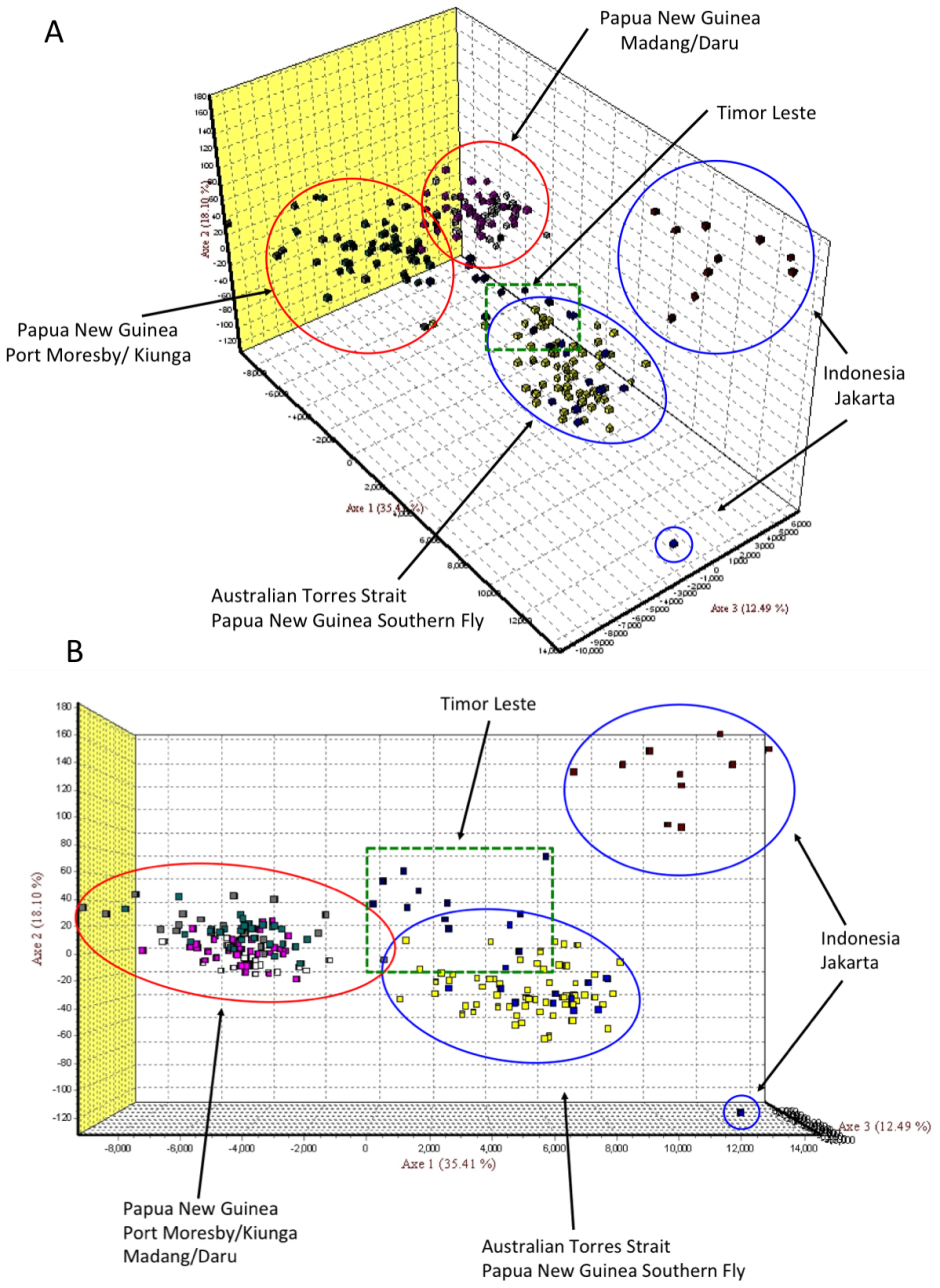
Factorial Correspondence Analysis (FCA) of 199 *Ae. albopictus* individuals. Each point represents one individual assessed for 13 microsatellites with their relative proximity to each other on the graph representing genetic relatedness. Panels A and B represent two perspectives of the graph.

The factorial correspondence analysis performed in GENETIX (Figure 4) supports the results of the STRUCTURE analysis. It clearly shows the close relationship between individuals from Daru Island and Madang, as well as between individuals from Port Moresby and Kiunga. Additionally, the introduced populations from the Torres Strait Islands and the southern Fly region are closely associated. The population with the greatest genetic affinities to the introduced population based on the FCA is Timor Leste, suggesting that the source of the introduction was more likely from the Indonesian region (where *Ae. albopictus* is common) than from the extant PNG populations, as had been previously hypothesized. The Jakarta population is relatively isolated on the FCA plot.

## Discussion

Detection of *Ae. albopictus* in southern Papua New Guinea (PNG) in 1988 and 1992 placed it only 150 km across the Torres Strait from mainland Australia. In 2004–05 it appeared on the Torres Strait Islands and we initially suspected a range expansion from PNG potentially driven by human adaptation to climate variability. The AusAID funded drought-proofing expansion of rainwater tanks and 200 L water containers into the southern Fly River region villages immediately adjacent to the Torres Strait was completed in 2002 as a response to climate variability in the region [33], [34]. Thus it was reasonable to hypothesize that the population historically extant in PNG had undergone a range expansion, initially into the southern Fly River region and subsequently into the Torres Strait Islands. The introduction of the species into the Torres Strait Islands was traced back to 2004 [29], at a date that appeared to correlate with the change in water management which had occurred a few years earlier.

Initially the mtCOI suggested that two genetically distinct populations were present in this region, providing the first piece of evidence that the invading population may not have originated from the population previously extant in PNG. Shared haplotypes between the southern Fly Region, the Torres Strait Islands and Indonesian populations provided the first clue as to where the invading population may have originated. Despite haplotype diversity being biased by the larval sampling method (which favors the collection of siblings of the same haplotype), the Torres Strait Island populations revealed four more haplotypes (a total of 10, Hd = 0.801) than the southern Fly River (6 haplotypes, Hd = 0.649). This difference in haplotype diversity may suggest that the initial introduction into the region started in the Torres Strait Islands from the Indonesian region, however more COI sequencing from the southern Fly region may be needed to clarify whether or not this is the case. Interestingly however, genetic diversity appears higher in the Torres Strait and Fly Region than in the Indonesian populations, possibly suggesting multiple introductions of closely related populations from different parts of Indonesia, or that the founding population was more genetically diverse than those sampled from the Indonesian region.

The mitochondrial DNA was informative at another level with the discovery that multiple *Ae. albopictus* mtDNA haplotypes (representing different females contributing to the population) were moving between islands. This suggests that attempts to eradicate the species from individual islands would likely be unsuccessful given the high potential for re-introductions. Indeed, this information has assisted Queensland Health – the regional state health authority – in its decision to move from the island eradication program implemented in 2006 to a *cordon sanitaire* in 2008, whereby surveillance and control was focused on the inner Torres Strait islands of Waiben, Muralug and Ngurupai (Thursday, Prince of Wales and Horn islands) adjacent to mainland Australia. In particular, Muralug and Ngurupai act as the major regional transport hubs and are thus the most likely staging point for the species' introduction onto the Australian mainland. This *cordon sanitaire* was breached in 2009 and *Ae. albopictus* now exists on Waiben and Ngurupia, less than 30 km from Australia's Cape York Peninsula. In 2010, *Aedes albopictus* larvae were collected from New Marpoon on mainland Australia's Cape York although no other individuals have been collected in this area since.

The 13 microsatellite loci reaffirmed the findings of the mitochondrial data that the introduced population was genetically distinct to the populations already present in PNG. As microsatellites evolve more rapidly than mitochondrial sequence data, they were more informative, resolving five genetically distinct populations in total with three historically extant populations in PNG that have experienced various levels of admixture and one distinct population encompassing the Torres Strait Islands and the southern Fly River region – the introduced population. Samples from Madang in northern PNG, as well as from Port Moresby on the southern Papuan Peninsula, appear to be admixtures, with individuals from Port Moresby being more similar to samples from Kiunga, and individuals from Madang more similar to the Daru Island population (Figures 3 and 4). Although Daru Island is proximal to the introduced population, material collected there on two separate occasions (1992 and 2008) was assigned with high probability to a separate population with close genetic affinities to the Madang material. Interestingly, the material collected from the Indonesian region (Timor Leste and Jakarta) forms a distinct population with apparent genetic affinities to the introduced population in the Torres Strait and southern Fly Region. Timor Leste revealed the highest genetic affinities in the FCA analysis and there are records of *Ae. albopictus* being present in Timor Leste dating to the 1920s [23]. Thus, our combined data strongly suggests that the introduction of *Ae. albopictus* into the Torres Strait and southern Fly River region came from the Indonesian region.

Since it now appears highly unlikely that the introduced population originated from PNG but rather came from the Indonesian region (west of the Torres Strait), it is conceivable that the introduction was driven by foreign fishing vessels that travelled from the Indonesian region, harboring *Ae. albopictus* specimens which then infested the islands of the Torres Strait and/or the southern Fly region. Indonesian/Macassan visitations to the coastline of northern Australia have a long history predating European settlement, and Indonesian fishermen have been known to illegally enter Australian waters more recently to fish for shark fin (Walker, J. Pers. Com.). This activity is reported to have peaked between 2005–07, during which time several hundred landings occurred where fuel, water, shark fin, fishing nets and lines were often cached. Many of these landings were associated with well-established camps that received multiple visits. Uninhabited islands in the Torres Strait were one focus for these activities and evidence gathered from apprehended vessels indicates that most shark boats (Type III - highly mobile, motorized vessels) carried large open-topped water drums of which a significant proportion harboured *Ae. aegypti* and *Ae. albopictus* larvae. There have been documented collections of various *Aedes* (*Stegomyia*) species, including *Ae. albopictus*, from illegal fishing vessels that were intercepted at the port of Darwin in the Northern Territory that clearly indicate that the mosquitoes' survival in smaller vessels is possible [56], [57].

With regard to the expansion and movement of *Ae. albopictus* in this region, populations on Daru Island (which adjoins the Torres Strait) appear to have been unaffected by this exotic invasion up until 2008 and follow-up collections are now warranted to determine if the introduced population has established there since. Despite Daru Island sitting geographically adjacent to the southern Fly River coastal region, its *Ae. albopictus* population seems to be clearly isolated from the introduced population. The occurrence of two genetically distinct populations adjacent to each other in the northern Torres Strait/PNG region can be best explained by their different jurisdictions. While the Torres Strait operates as part of Australia, Daru Island is politically part of PNG and its function as the international customs clearance station into Western Province means that it encounters different incoming and outgoing transport movements.

The movement of *Ae. albopictus* appears to be extensive in the Australasian region, particularly where the Torres Strait Islands' junction separates New Guinea and mainland Australia. With the maximum reported flight range of *Ae. albopictus* being 1 km [58], movement between the Torres Strait's islands has most likely taken place via human-mediated transport. Australia has an oceanic border with PNG and the Torres Straits, but unlike most other international countries, it has no clearly marked frontier with border policing or customs controls. Thus relatively free movement occurs between PNG and the Torres Straits with approximately 5,000 international shipping movements per year [59], and countless domestic movements that would likely shuttle *Ae. albopictus* between the islands and back and forth from the southern Fly region's villages. This movement is sanctioned under *The Torres Strait Treaty (Miscellaneous Amendments) Act 1984* that allows for cross-border movement for trade, fishing, and family gatherings or for seeking medical attention without the need for customs protocols [60] –all of which may compromise quarantine in the region. The primary mode of transport between PNG villages and the Torres Strait Islands is via open-topped, outboard-motor-powered boats. In addition to sheltered harborage sites for adult mosquitoes, these vessels contain fresh water – both within drums for human consumption, and where rainwater has collected – which in turn provides oviposition sites and larval habitats for container-inhabiting species such as *Ae. albopictus*.

Given its current location, its mobility and its phenotypic fondness for containers, *Ae. albopictus* is more than likely to arrive in a town or city on the Australian mainland via human transport. Due to the intrinsic ecological plasticity in both larval habitat (both natural and human-made) and host feeding patterns [3], it will likely be able to move between urban and sylvan habitat, and control will be extremely challenging once it enters the latter. Its effect on native virus vector systems in Australia represents an unknown risk both to humans and to native and domestic animals. However, its cool climate tolerant biology and plasticity will certainly present new risks for dengue and chikungunya transmission in summer throughout most of the Australian region [27], [30].

*Aedes albopictus's* particular biology permits its container inhabiting ecological niche to once again facilitate its global expansion. It is important to consider it's potential to rapidly exploit the outcomes of any socio-economic or policy-driven interventions, such as the recent and dramatic expansion of domestic rainwater tanks throughout Australian urban regions as a drought-proofing adaptation to observed and forecasted climate change [30]. Although this adaptation did not ultimately explain the range expansion of the species on Australia's northern doorstep, it will nonetheless provide a valuable niche in the landscape, which may augment this vector's existence across Australia's urban regions. In considering its eventual arrival, the public health risks associated with arboviruses meet the possibility of substantial daytime nuisance biting that will also negatively impact Australia's urban alfresco lifestyle.
